# Supplementary material for: Association between oral microbiome and seven types of cancers in East Asian population: a two-sample Mendelian randomization analysis
Source: Front Mol Biosci. 2023 Nov 21;10:1327893. doi: 10.3389/fmolb.2023.1327893 (PMC10702768; doi:10.3389/fmolb.2023.1327893)
Supplement: Supplementary file 1 [file DataSheet1.ZIP › Supplementary Materials/MR plots of saliva/saliva═╝/colorectal cancer/pheno.2883_to_colorectal cancer_forest.pdf]

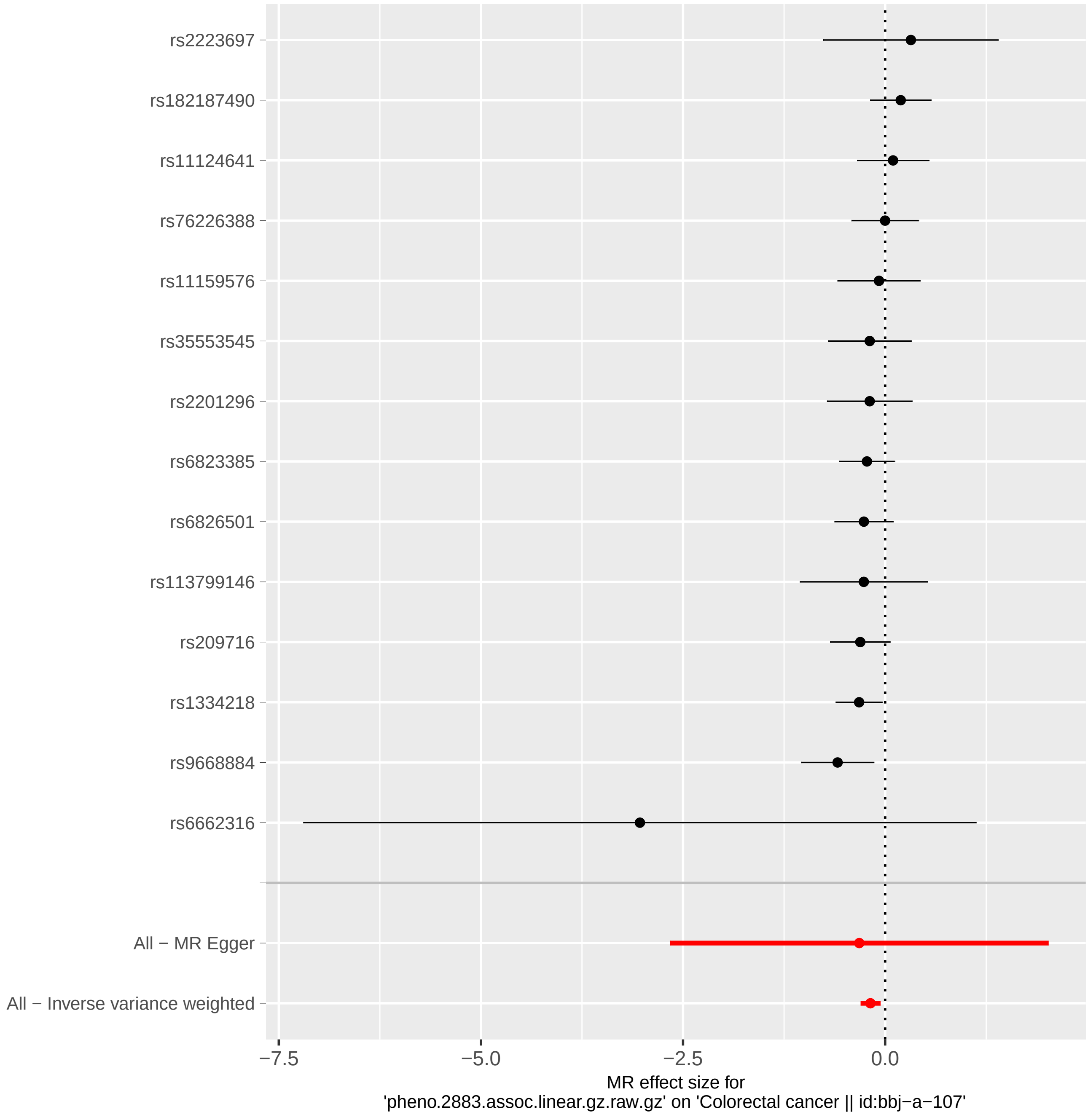
rs2223697
rs182187490
rs11124641
rs76226388
rs11159576
rs35553545
rs2201296
rs6823385
rs6826501
rs113799146
rs209716
rs1334218
rs9668884
rs6662316
All − MR Egger
All − Inverse variance weighted
−7.5
−5.0
−2.5
0.0
MR effect size for
'pheno.2883.assoc.linear.gz.raw.gz' on 'Colorectal cancer || id:bbj−a−107'
